# Profile of diseases prevalent in Datia District of Madhya Pradesh, India

**DOI:** 10.3389/fpubh.2022.926000

**Published:** 2022-09-29

**Authors:** Nalini Mishra, Pradip V. Barde, Sarvesh Awasthi, Archana Kumawat, Rajesh Gaur, Pushpendra Singh, Suyash Srivastava, Aparup Das

**Affiliations:** ^1^ICMR-National Institute of Research in Tribal Health, Jabalpur, India; ^2^Model Rural Health Research Unit, ICMR-NIRTH, Jabalpur, India; ^3^Government Medical College, Datia, India

**Keywords:** disease profiling, Datia District, TB, lifestyle diseases, socio economic conditions, health systems

## Abstract

**Background and objectives:**

Exploring the disease profile in a defined area helps policymakers to understand local health issues. It is essential to collect such information in countries, like India, which have a wide range of socioeconomic, geographic, and cultural diversity. Madhya Pradesh is the second largest state of India and has diversified populations living in urban, rural, and tribal areas. In this study, we performed a hospital record-based analysis to find out the status of different diseases in various outpatient departments (OPDs) of the District Hospital where patients from both rural and urban areas are treated.

**Materials and methods:**

The retrospective data was collected from medical records available for the period September 2018 to September 2020. These datasets were analyzed to determine the most common presentations among patients.

**Results and conclusions:**

A total of 138,756 records of patients were available for this study, whose department-wise analysis revealed that most records were related to respiratory tract infection, superficial dermatophytosis, anemia among women, suppurative otitis media, and pneumonia. This study provides a broad overview of the overall health issues of people living in rural and urban areas of Datia. However, a major limitation of the study was that other correlations with age and gender were not established due to the absence of such information. Nonetheless, these findings will help policymakers and researchers to set the agenda for interventions and set goals for achieving better health for all people including those living in rural and tribal regions.

## Introduction

Disease profiling plays an important role in making health systems stronger, and can help in allocating resources proportionate to the relative distribution of a disease. Lifestyle habits, nutrition, conditioning, access to health services, and literacy affect the lives of individuals directly or indirectly. Vital risk factors for various diseases that affect health can include environmental and ongoing developmental activities in a region. Besides the communicable disease outbreaks, endemic diseases such as malaria, tuberculosis (TB), acquired immunodeficiency syndrome (AIDS), and neglected tropical diseases (NTDs), such as leprosy, lymphatic filariasis, dengue, chikungunya, are of particular concern and challenge public health infrastructure, requiring a high level of preparedness for early detection and rapid action (1). According to reports, non-communicable diseases (NCDs) are now the leading cause of morbidity and mortality in the country, contributing to 60% of all deaths (1, 2). Four diseases, namely cancer, cardiovascular disease, chronic pulmonary diseases, and diabetes, caused nearly 80% of all deaths (3). Lifestyle habits like use of tobacco and alcohol, unhealthy diet, and lack of physical activities are four common risk factors for all these diseases. Another important health challenge is antimicrobial resistance which must be tackled with all readiness (4).

**Figure 1:**
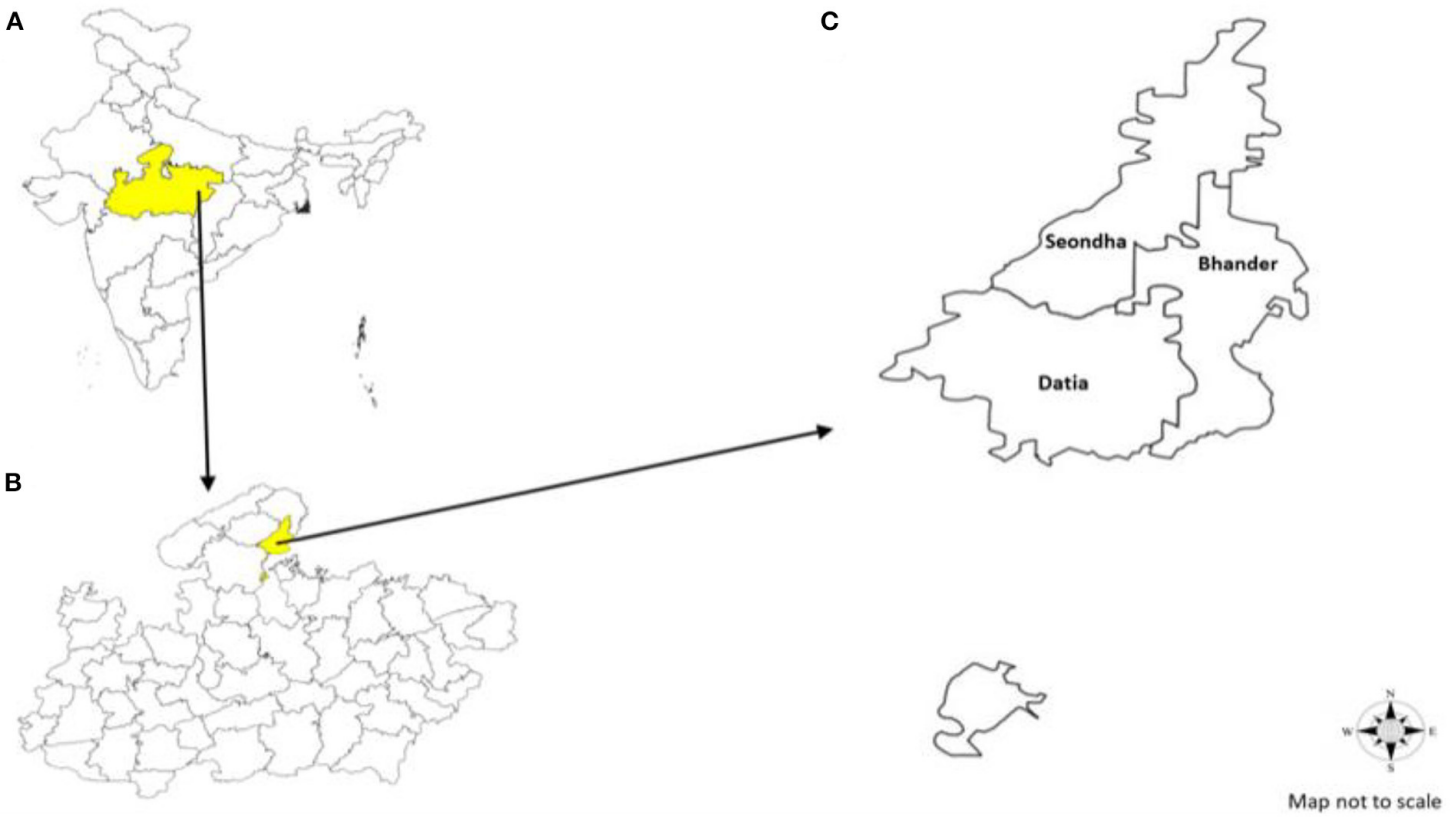
**(A)** Map of India highlighting Madhya Pradesh State. **(B)** Map of Madhya Pradesh highlighting Datia District. **(C)** Map of Datia District with administrative blocks of the district.

Datia is one of the smallest districts of Madhya Pradesh (MP) located in the northern part of the state. It is located 25.67 latitude and 78.46 longitude (Figure 1) and it is situated at an average elevation of 249 m above sea level. To our knowledge, there has been no disease-profiling study carried out in this district, except for one study which indicated the prevalence of filariasis (5). Due to very limited information available on this subject, it is difficult to know which diseases and/clinical presentations are more common in the area. Therefore, this study aimed to determine the disease profile and relative magnitude based on the clinical presentations reported at the District Hospital, which is a government medical facility where patients from the entire district are referred and treated. Such types of data will help to identify important facts about the disease profile to be considered for the proposed research works for the Model Rural Health Research Unit (MRHRU), Datia, and will also enable other researchers and medical authorities to undertake research in different priority areas depending upon the health needs and presentations of the people living in Datia District and the adjoining areas.

## Materials and methods

### Study area, study population, and study sites

Datia is a relatively small and predominantly rural district situated in the northern Bundelkhand region of Madhya Pradesh (Figure 1). It covers an area of 2,959 km^2^, which is 0.94% of the total area of the state. According to Census 2011, there are 631 villages in total, of which 586 villages are inhabited and 45 villages are uninhabited. Datia District has a population of 786,754 (420,157 are male and 366,597 are female). The reported tribe population of the district is 15,061 (1.91%) out of which 7,870 (52.25%) are male and 7,191 (47.75%) are female. The average literacy rate of the district is 72.6%, and the sex ratio is 873 female inhabitants per 1,000 male inhabitants (6).

### Data collection and analysis

This is a retrospective study analyzing the data of the patients who visited the District Hospital Datia from September 2018 until September 2020. Electronic medical records were used for this analysis. The data were collected from various departments, namely general medicine; dermatology; ear, nose, and throat (ENT), obstetrics and gynecology; pediatrics; pulmonary medicine (complaints relating to the chest, including TB); psychiatry; community medicine; and surgery. Diagnostic criteria for diagnosis of anemia and gestational diabetes were according to the WHO (7). The study was approved by the Institutional Ethics Committee of ICMR-National Institute of Research in Tribal Health, Jabalpur, *via* letter no. NIRTH/IEC/01/31/2021. All sets of data were entered and analyzed using Microsoft Office Excel (2007). Descriptive analysis was done to determine a quantitative estimation of different clinical presentations reported by the patients in the district.

## Results

A total number of 138,756 patients visited during the study period. The data was analyzed department wise.

**Figure 2:**
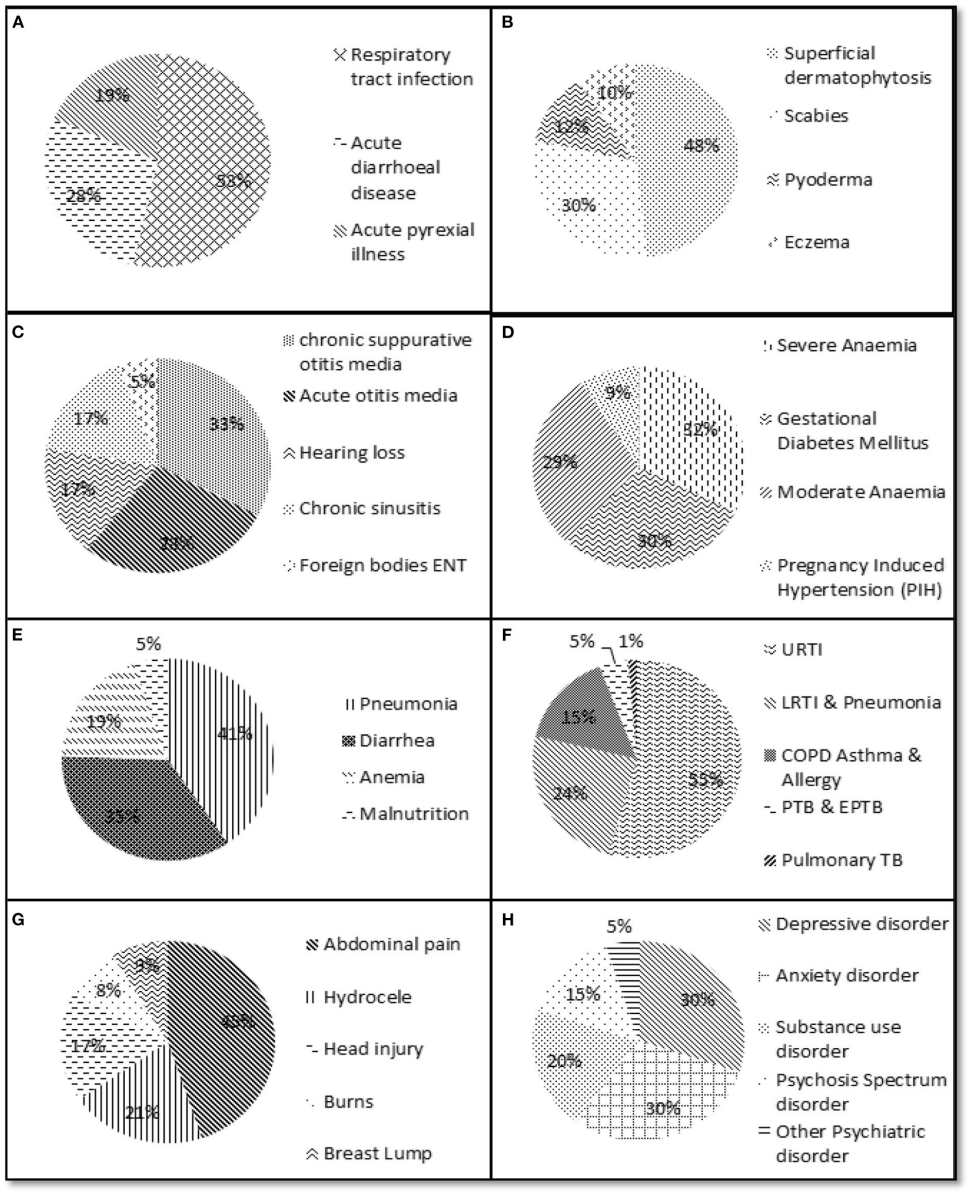
Depiction of percentage of important clinical presentations/diseases reported to different departments. **(A)** General medicine department. **(B)** Dermatology department. **(C)** Ear, nose and throat department. **(D)** Obstetrics and gynecology department. **(E)** Pediatrics department. **(F)** Pulmonary medicine department. **(G)** Surgery department. **(H)** Psychiatry department.

The data of hospital records indicated that out of the 31,550 records of the patients who visited the department of General Medicine (Figure 2A), a majority (16,890, 53%) had reported respiratory tract infection followed by acute diarrheal diseases (*n* = 8,800, 27.9%). In the department of dermatology (Figure 2B), a total of 35,460 patient records were available, of which *n* = 17,089 (48.2%) had complaints of superficial dermatophytosis (48%). In the ear, nose- and throat (ENT) department, there were records of 21,400 visits of patients, of which 33% were provided with treatment or a prescription for chronic suppurative otitis media (Figure 2C). In the obstetrics and gynecology department, there were records corresponding to 1,433 patient visits, of which severe anemia (461, 32.1%) and gestational diabetes mellitus (427, 29.7%) were more common than other presentations (Figure 2D). In the department of pediatrics (Figure 2E), a total of 3,255 patient records were available, of which a majority recorded pneumonia and diarrhea (41 and 35%, respectively). A total number of 24,504 patients were reported in the department of pulmonary medicine, of which a majority (*n* = 13,342, 54.4%) had symptoms of upper respiratory tract infection (Figure 2F). The surgery department had a record of 20,281 visits of patients, most of which were related to abdominal pain (*n* = 9,092/20,281 = 44.8%; Figure 2G). In the department of psychiatry, a total of 873 patient records were available, of which 524 (60%) reported depressive disorders such as depression and anxiety (Figure 2H).

## Discussion and conclusions

Knowledge of the prevalent clinical presentations and diseases, and the factors associated with them over a period of time is of paramount importance to plan health infrastructure and interventions accordingly. The majority of the Indian population is dependent on medical services provided at government-funded facilities (8). The District Hospital is the biggest government health facility that is accessible and affordable for all sections of society, and where people from urban areas as well as villages visit or are referred for diagnosis or treatment. However, there are no data currently available regarding the disease profile of Datia District. Therefore, in the present study, we carried out retrospective data analysis of the hospital records.

In this study, we quantitated the clinical presentations for which treatment or diagnosis was sought by patients. Cases from PHCs/CHCs in the entire district are also referred for diagnostics and treatment at the District Hospital, as it is relatively better equipped to handle various types of diseases, including their diverse clinical manifestations of varying severity. Hence, the District Hospital receives a good representation of various types of significant health issues faced by the inhabitants of the entire district. Therefore, we chose to analyze the data retrospectively to generate useful information about disease profiling in Datia District.

We identified that respiratory tract infections have become a significant health problem. As a result of rapid changes and development, outdoor air pollution has been increasing, for example, due to ongoing constructional works (building of national highways, malls, shops, etc.). This has increased a variety of pollutants, dust particles, and particulate matter in the environment (9), hence most patients were found to have respiratory tract-related problems. Detailed investigations in this regard are necessary to determine the appropriate measures to minimize the causes of such problems. Although we were not able to specifically determine the cases of tuberculosis from this data, it is noteworthy that several neighboring districts (such as Shivpuri and Sheopur, with a significant tribal population) have hyper-endemic regions with a very high prevalence of tuberculosis (1,518 per 100,000 population), and the annual risk of tuberculosis infection (ARTI) has been estimated to be 3.9% in these tribal populations (10, 11).

Since many of these tribal populations, particularly those belonging to the Saharia tribe, are also known to undertake short-term seasonal migration (as laborers at the time of the crop harvest) in neighboring regions, it is likely that tuberculosis is also an important contributor to the cases of respiratory infections reported at the District Hospital. It is also consistent with the well-established fact that tuberculosis among members of the Saharia tribe is a significant public health concern in the Chambal division of the state. However, a more detailed laboratory or clinical study would be needed to confirm this. Nonetheless, there are several commonly observed factors that can compromise the overall lung health of a person. such as improper ventilation inside houses, use of smoke *Chulhas* for cooking inside the house, and the practice of sharing items used for smoking tobacco (such as *Bidi* and *Chilums*).

Diseases or inflammation that affect the organs in the abdominal area can cause abdominal pain. The microbiological and biochemical quality of water used by the majority of the population for cooking and drinking purposes is often below the recommended levels. This is due to many reasons, such as the shortage or non-availability of fresh water sources for most of the population. Abdominal pain was the most common complaint in the surgery department. We do not have all the details about the exact causes of abdominal pain. However, the water in Datia has a high level of salts and minerals, and therefore use of this water for drinking and cooking may increase the chances of stone formation, and it is possible that advanced cases of such presentations report to the surgery department. In Datia District, water is usually supplied by water tankers and then stored in tanks for days. This might be a contributing factor to abdominal pain caused by diarrhea, stomach infections, or constipation. Various bacterial, viral, or parasitic infections can affect the stomach and intestine and cause significant abdominal pain.

As stated earlier, Datia is predominantly a rural setting, and many people are engaged in outdoor farming activities and rearing animals. Thus, various diseases, such as waterborne diseases (e.g., typhoid), vector-borne diseases (e.g., malaria, filariasis, dengue, chikungunya), and diseases spread through contact with contaminated objects (such as fomite-borne infectious diseases) (e.g., dermatophytosis), could have their origins (such as contact with farm animals, cattle reared for milk, and the use of cow dung as manure and fuel for cooking) in the socio-economic practices and demographic factors of the population present there. The most common problem observed in the obstetrics and gynecology department was anemia; pregnant women are often not able to eat a balanced diet, and this is reflected in the poor nutritional status of the population. Thus, the presentations observed in this study were mostly related to poor hygiene and ventilation, overcrowding, and poor nutritional status (2).

The strength of this study was a large sample size and coverage of various diseases of different departments, as this data spans 2 years (2018–2020). Therefore, to a large extent, it could reveal the characteristics of disease in this district's population. During this time, the District Hospital was administratively attached to the mentorship of the Government Medical College in Datia District. The Model Rural Health Research Unit (MRHRU) Datia also began its work at the Government Medical College, Datia, during this period and aimed to gather information which would be useful and timely for planning further research work and projects considering the pressing health issues of the population living in Datia.

The limitations of this study were that we were not able to retrieve information about the age and sex of the patients, and we could not determine the seasonal trends in these diseases. This study was performed retrospectively using anonymized data made available from the District Hospital for determining the relative magnitude and diversity of various health issues. In the absence of this information, we were not able to explore any correlations between different diseases or presentations and other variables such as age, sex, etc. Thus, it is recommended that future studies should collect information on these variables as well, so that there can be a deeper insight into the disease profile.

Since this kind of survey had not been done before in this study setting, it provided some background on the overall disease status of the population. It is the first study of this type, representing data of 2 years from Datia District, and has identified key areas for future research. However, we have only been able to provide some possible speculation about underlying factors that would need to be explored in more detail in future studies. Health authorities can still take this information into account to identify priority areas, and suitable preparations or interventions can be planned accordingly. This study also identifies problematic areas to be worked upon, for example: improvement in nutrition, anemia (especially among pregnant women, as their poor nutritional status leads to several health issues not only for them but also for their children), gestational diabetes mellitus, pregnancy-induced hypertension in women, causes of abdominal pain, dermatophytosis, etc. In a similar study in the Gorakhpur Health and Demographic Surveillance Site, the overall prevalence of non-communicable disease (NCD) multimorbidity was found to be 1.8%. This study also found that those with an NCD have out-of-pocket expenditure on health issues that is four times higher than those without an NCD (12).

Presently, the concept of One Health is being widely adopted, as health is influenced by many factors (13), which may be broadly classified into five “determinants of health,” namely: genetics, behavior, physical and environmental influences, medical care, and social factors. These five determinants are interconnected. There needs to be more research on the factors that are associated with various diseases or clinical presentations described in this study. This will also help to calculate the optimal human resources at medical facilities to enable better allocation of resources, including manpower. Such studies might help to advance the provision of medical facilities required and could provide useful preliminary data to help strengthen the requisite level of medical research.

One of the biggest focuses in the last few years has been to address equity, because one of the purposes of public health is to enable health equity by offering affordable and accessible health services to all, including rural and tribal populations. In conclusion, tuberculosis, anemia, gestational diabetes mellitus, dermatophytosis, water-quality related diseases, and depression are the biggest health issues in Datia District. Therefore, researchers and policymakers should take this into account during the planning and implementation of relevant health policies and services in these areas. In this regard, priority should be given to investigating the barriers to equitable access to health infrastructure by integrating equity aims in policies and programs.

## Data availability statement

The raw data supporting the conclusions of this article will be made available by the authors, without undue reservation.

## Ethics statement

The studies involving human participants were reviewed and approved by Institutional Ethics Committee, ICMR-National institute of research in Tribal Health. Written informed consent to participate in this study was not required as it was a retrospective study.

## Author contributions

NM: wrote the manuscript, experimentation, and analyzed the data. PB: experimentation, investigator, and edited the manuscript. AK: done experimentation and compiled the data. RG: research design and data collection. SA: analyzed the data. PS and SS: editing and compiling. AD: research design and editing. All authors contributed to the article and approved the submitted version.

## Conflict of interest

The authors declare that the research was conducted in the absence of any commercial or financial relationships that could be construed as a potential conflict of interest.

## Publisher's note

All claims expressed in this article are solely those of the authors and do not necessarily represent those of their affiliated organizations, or those of the publisher, the editors and the reviewers. Any product that may be evaluated in this article, or claim that may be made by its manufacturer, is not guaranteed or endorsed by the publisher.
